# Mangosteen peel extract (*Garcinia mangostana* L.) as protective agent in glucose-induced mesangial cell as *in vitro* model of diabetic glomerulosclerosis

**DOI:** 10.22038/IJBMS.2018.29349.7094

**Published:** 2018-09

**Authors:** Wahyu Widowati, Dian Ratih Laksmitawati, Teresa Liliana Wargasetia, Ervi Afifah, Annisa Amalia, Yukko Arinta, Rizal Rizal, Tri Suciati

**Affiliations:** 1 Faculty of Medicine, Maranatha Christian University, Bandung 40164, West Java, Indonesia; 2 Faculty of Pharmacy, Pancasila University, Jakarta Selatan 12630, DKI Jakarta, Indonesia; 3 Aretha Medika Utama, Biomolecular and Biomedical Research Center, Bandung 40163, West Java, Indonesia; 4 School of Pharmacy Bandung Insitute of Technology, Bandung 40132, West Java, Indonesia

**Keywords:** Fibronectin, Glomerulosclerosis, Garcinia mangostana, Mesangial cell, Transforming growth factor-β1

## Abstract

**Objective(s):**

This study aims to evaluate the activity of mangosteen peels extract (MPE) as protection agent on induced-glucose mesangial cells (SV40 MES 13 cell line (*Glomerular Mesangial Kidney, Mus Musculus*)).

**Materials and Methods:**

MPE was performed based on maceration method. Cytotoxic assay was performed based on MTS (3-(4,5-dimethylthiazol-2-yl)-5-(3-carboxymethoxyphenyl)-2-(4-sulfophenyl)-2H-tetrazolium) method, while the level of TGF-β1 (Transforming growth factor-β1) and fibronectin in glucose-induced mesangial cells were assayed and determined using ELISA KIT.

**Results:**

In viability assay, MPE 5 and 20 µg/ml has the highest activity to increase cells proliferation in glucose-induced mesangial cells at 5, 10, and 15 days of incubation in glucose concentration (5 and 25 mM) (*P<*0.05). In inhibitory activity of TGF-β1 and fibronectin level, MPE 5 µg/ml (glucose-induced 5 mM) show the lowest level compared to positive control and other treatments (*P<*0.05).

**Conclusion:**

MPE can increase cell proliferation in glucose-induced mesangial cells and significantly reduce the level of TGF-β1 and fibronectin. MPE activity has correlates to inhibit the diabetic glomerulosclerosis condition and may increase mesangial cell proliferation.

## Introduction

According to the International Diabetes Federation, the prevalence of diabetes in the world is estimated to increase from 285 million persons to 439 million in 2030 (1). Diabetic is known to be a leading cause of end-stage renal failure (2). All forms of diabetes are characterized by hyperglycemia (3). Hyperglycemia is the primary pathogenic factor for diabetic nephropathy (DN) (4). Through multiple mechanisms, diabetic nephropathy can develop to end-stage kidney disease but none is as important as the gradual, inexorable scarring of the renal glomerulus, known as glomerulosclerosis (5). Glomerulosclerosis is diabetic nephropathy caused by accumulation of extracelullar matrix (ECM) proteins in mesangial interstitial space, resulting in fibrosis manifested by either diffuse or nodular changes (6). One of the most common matrix protein detected is fibronectin (5). Several studies also found that hyperglycemia induces reactive oxygen species (ROS) production in mesangial cells that up-regulates Transforming Growth Factor Beta (TGF-β) involved in ECM accumulation (7, 4).

During the centuries, natural substances from plant has been widely used for treating and preventing some various diseases. Most of these natural substances were studied, isolated, and converted into modern medicine (8). These natural substances and its compounds would be promising alternative for therapeutic in respect of low cost, highly compatible with dietary intake and no harmful effects inside the human body (9). *Garcinia mangostana *Linn. or commonly known as mangosteen is a tropical fruit from South East Asia (10). Not only known from its flesh as a dessert, the peels of mangosteen are also known to treat various infectious diseases of skin and wounds, diarrhea, dysentery, cholera and have anti-inflammatory (11-13), anticancer potency (14). The peels of mangosteen are reported to be rich of phenolic compounds with potential applications as therapeutic agents such as phenolic acids (15), tannins (16), xanthones and anthocyanins (17, 10, 13). From these various activities, the potential of mangosteen peels against atherosclerosis is thought to be derived from antioxidant (18), antiagreggation (19), antiobesity (20, 21, 22) anti-inflammatory properties (13). Mangosteen peel extract (MPE) containing many active compounds are expected to inhibit and retard progression of diabetic glomerulosclerosis into renal chronic disease. This research was conducted to evaluate the potential of MPE and its component α-mangostin (AM) as protective agent in glucose-induced mesangial cell as *in vitro* model diabetic glomerulosclerosis.

## Materials and Methods

***Plants extract preparation***

*G. mangostana *L. was collected from Cisalak-Subang, West Java, Indonesia plantation and identified by a staff of Herbarium of Department of Biology, School of Life Science and Technology, Bandung Institute of Technology, Bandung, West Java, Indonesia. The mangosteen peels were collected, chopped, and kept in drier tunnel service. Extraction was performed based on maceration method using distilled ethanol 70% as the solvent for collecting *G. mangostana *L. peel extract (MPE) (13,19).

***Viability assay***

The Glomerular Mesangial Kidney, *Mus musculus *(SV40 MES 13 ATCC ® CRL-1927™) was obtained from Biomolecular and Biomedical Research Center, Aretha Medika Utama, Bandung. The viability assay was performed using MTS (3-(4,5-dimethylthiazol-2-yl)-5-(3-carboxymethoxyphenyl)-2-(4-sulfophenyl)-2H-tetrazolium) Proliferation Assay Kit (Abcam, ab197010). In brief, 5x10^3 ^cells per well in F12-K medium (Gibco, 21127022) and DMEM (Gibco, 11995065), 10% fetal bovine serum (FBS, Gibco, 10270106), and 1% antibiotic-antimycotic (Gibco, 1772653), 1% HEPES (Sigma Aldrich, 1002184736) were cultured in 96 well plate (Corning, 3596) and incubated at 37 ^°^C, 5% CO_2 _for 24 hr. Then, medium replaced with 180 μl of fresh medium, 20 μl of MPE (5 µg/ml and 20 µg/ml), AM (20 µM and 80 µM) and DMSO 10% were added in triplicate and the plates were incubated at 37 ^°^C, 5% CO_2 _for 24 hr. Untreated cells were served as the control. Briefly, 20 μl MTS was added to each well. Then, the plate was incubated at 37 ^°^C, 5% CO_2 _for 4 hr. The absorbance was measured at 490 nm using Multiskan GO plate reader (Thermo Scientific, U.S.A) (23, 24, 13).

***Glucose-induced mesangial cells for proliferation assay***

Briefly 5x10^3 ^cells/well of SV40 MES 13 cells were platted in 96-well plate with 200 μl growth medium and incubated at 37 ^°^C, 5% CO_2 _for 24 hr. The medium was discarded then added with 180 μl glucose-induced medium (5 mM, 20 mM, 50 mM, and 115 mM) 20 μl MPE (5, 20 µg/ml) and 20 μl AM (20, 80 µM). After that, the cells were incubated at 37 ^°^C, 5% CO_2 _for 14 days. Proliferation was measured every 2 days using MTS Proliferation Assay Kit (Abcam, ab197010). The absorbance was measured at 490 nm using Multiskan GO plate reader (Thermo Scientific, U.S.A) to calculate the percentage of cell mortality (25, 13).

**Figure 1:**
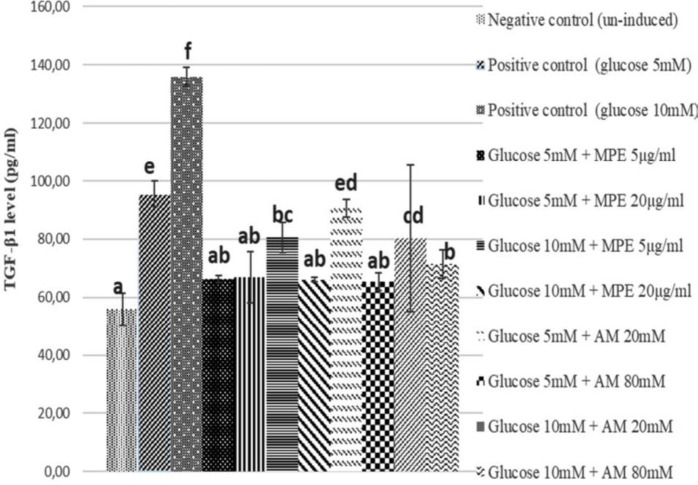
TGF-β1 level of glucose-induced SV40 MES 13 cells treated with mangosteen peel extract and α-mangostin

*Data were analyzed with ANOVA and Duncan post hoc. The different letter (a,ab,b,bc,cd,de,e,f) show significant differences among treatments (*P* <0.05).

Each samples were done in triplicate. Negative control was un-induced mesangial cells, positive control I was glucose 5 mM-induced cells, positive II was glucose 10 mM-induced cells.

**Figure 2:**
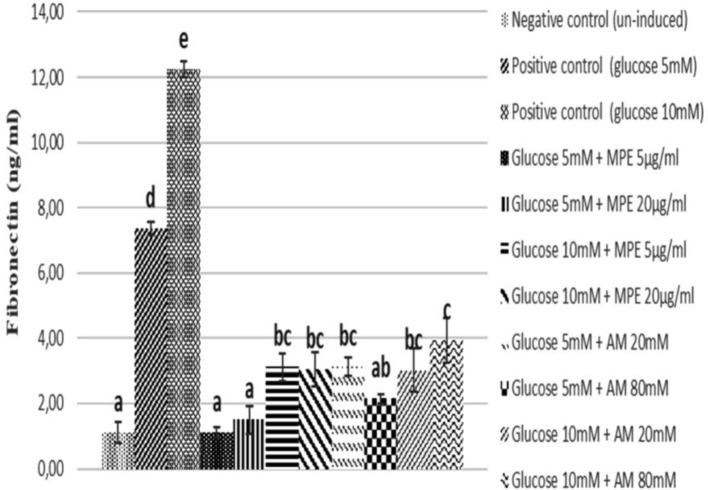
Fibronectin level of glucose-induced SV40 MES 13 cells treated with mangosteen peel extract and α-mangostin

*Data were analyzed with ANOVA and Duncan *post hoc test*. The different letters (a, ab, bc, c, d, e) show significant differences among treatments (*P<*0.05). Each samples were done in triplicate. Negative control was un-induced mesangial cells, positive control I was glucose 5 mM-induced cells, positive II was glucose 10 mM-induced cells

**Table 1:** Effect of mangosteen peel extract and α-mangostin toward cells proliferation in glucose-induced mesangial cells at 5 days of incubation

Concentration	Glucose Concentration			
	0 mM	5 mM	25 mM	125 mM
Control	100.00±5.84^a^	100.00±5.84^a^	100.00±5.84^a^	100.00±5.84^bc^
Positive Control		96.71±0.91^a^	84.78±1.71^a^	74.92±1.30^a^
MPE 20 µg/mL	136.32±28.75^ab^	207.70±7.02^b^	162.18±8.56^bc^	121.04±12.52^c^
MPE 5 µg/mL	153.94±26.71^b^	214.61±3.19^b^	199.38±19.80^c^	121.20±12.43^c^
AM 80 mM	113.39±26.71^ab^	175.18±42.90^b^	153.46±5.35^c^	99.28±2.04^bc^
AM 20 mM	143.33±3.17^ab^	159.76±25.46^b^	193.13±24.44^b^	98.30±7.75^b^

* Data were served in average ± standard deviation. Different superscript letters in the same column of 0 mM (a,ab,b), 5 mM (a,b), 25 mM (a,b,bc,c), 125 mM (a,b,bc,c) glucose concentration show significant differences among treatments per induction (*P*<0.05) analyzed using ANOVA and Duncan post hoc test.

**Table 2:** Effect of mangosteen peel extract and α-mangostin toward cells proliferation in glucose-induced mesangial cells at 10 days of incubation

Concentration	Glucose Concentration			
	0 mM	5 mM	25 mM	125 mM
Control	100.00±8.57^a^	100.00±8.57^ab^	100.00±8.57^bc^	100.00±8.57^c^
Positive Control		94.64±0.43^a^	86.84±1.50^a^	73.26±2.25^a^
MPE 20 µg/mL	199.39±20.50^b^	127.68±3.33^d^	122.02±3.10^d^	91.93±0.15^bc^
MPE 5 µg/mL	198.59±15.13^b^	110.91±6.34^bc^	111.26±5.52^cd^	89.79±0.22^b^
AM 80 mM	145.65±8.69^ab^	115.64±0.64^cd^	101.14±0.53^bc^	91.88±0.34^bc^
AM 20 mM	174.85±40.73^b^	101.15±0.27^ab^	98.53±1.61^ab^	91.49±1.61^bc^

* Data were served in average ± standard deviation. Different superscript letters in the same column of 0 mM (a,ab,b), 5 mM (a,ab,bc,cd,d), 25 mM (a,ab,bc,cd,d), 125 mM (a,b,bc,c) glucose concentration show significant differences among treatments per induction (*P*<0.05) analyzed using ANOVA and Duncan post hoc test.

**Table 3:** Effect of mangosteen peel extract and α-mangostin toward cells proliferation in glucose-induced mesangial cells at 15 days of incubation

Concentration	Glucose Concentration			
	0 mM	5 mM	25 mM	125 mM
Control	100.00±7.75	100.00±7.75^ab^	100.00±7.75^b^	100.00±7.75^b^
Positive Control		76.89±2.13^a^	70.94±1.80^a^	54.12±4.18^a^
MPE 20 µg/mL	116.16±4.04	113.38±5.10^b^	97.85±0.58^b^	59.11±13.86^a^
MPE 5 µg/mL	118.12±3.90	104.60±15.85^ab^	83.32±2.17^ab^	68.42±8.11^a^
AM 80 mM	122.34±12.65	100.17±11.03^ab^	78.08±4.61^a^	62.08±8.75^a^
AM 20 mM	121.98±16.65	104.83±13.32^ab^	72.85±11.71^a^	70.31±5.27^a^

* Data were served in average ± standard deviation. Different superscript letters in the same column of 5 mM (a,ab,b), 25 mM (a,ab,b), 125 mM (a,b) glucose concentration show significant differences among treatments per induction (*P*<0.05) analyzed using ANOVA and Duncan post hoc test.

***Quantification of TGF-β1 level***

The quantitative determination of TGF-β1 level in the cell-free supernatant was performed using Rat TGF-β1 ELISA Kit (ElabSci E-EL-R0084) based on manufactured protocol. Briefly, 100 µl of standard, blank, and sample solution was added into each well then sealed and incubated for 90 min at 37 ^°^C. After treating with MPE and AM, the cell-free supernatant was served as the sample. The glucose-induced mesangial cell free supernatant without extract and compounds were used as positive control. The normal cell or untreated cell was used as negative control. Subsequently, the liquid of each well was discarded and 100 µl biotinylated detection Ab was added and then incubated for an hr at 37 ^°^C. Then the liquid was discarded and the plate was washed three times using 200 µl wash buffer. HRP conjugate (100 µl) was added and incubated for 30 min at 37 ^°^C. The liquid was discarded again and the plate was washed five times using 200 µl wash buffer. Substrate reagent (90 µl) was added and incubated for 15 min at 37 ^°^C. Stop solution (50 µl) was added and the absorbance was read at 450 nm using Multiskan GO plate reader (Thermo Scientific, U.S.A) (26). 

***Quantification of fibronectin level***

The quantitative determination of fibronectin level in the cell-free supernatant was performed using Rat FN (Fibronectin) ELISA Kit (ElabSci E-EL-R0578) based on manufactured protocol. Briefly, 100 µl of standard, blank, and sample solution was added into each well then sealed and incubated for 90 min at 37 ^°^C. The cell-free supernatant, after treated with MPE and AM, were served as the sample. The glucose-induced mesangial cell free supernatant without extract and compounds were used as positive control. The normal cell or untreated cell was used as negative control. Subsequently, the liquid of each well was discarded and 100 µl of biotinylated detection Ab was added then incubated for an hour at 37 ^°^C. The liquid was discarded again and the plate was washed three times using 200 µl wash buffer. HRP conjugate (100 µl) was added and incubated for 30 min at 37 ^°^C. The liquid was discarded again and the plate was washed five times using 200 µl wash buffer. Substrate reagent (90 µl) was added and incubated for 15 min at 37 ^°^C. Stop solution (50 µl) was added and the absorbance was measured at 450 nm using Multiskan GO plate reader (Thermo Scientific, U.S.A) (27). 

***Statistical analysis***

The data was analyzed using SPSS 16 (SPSS Inc., Chicago, IL, USA) to perform one-way ANOVA to verify the results of different treatments and Duncan *post hoc* was used to validate significant differences for all treatments (*P*<0.05). The results are displayed as means±standard deviation.

## Results

***Viability assay***

The viability of glucose-induced SV40 MES 13 cells during 5 days of incubation time treated with MPE (5, 20 μg/ml) and AM (20, 80 μM) can be seen in Table 1. Cell treated with MPE 5 µg/ml showed the highest cell proliferation. This indicated that MPE 5 µg/ml has a good viability and activity to increase cell proliferation in glucose-induced mesangial cells at 5 days of incubation compared to other treatments.

The viability of glucose-induced SV40 MES 13 cells treated with MPE (5, 20 μg/ml) and AM (20, 80 μM) during 10 days of incubation time can be seen in Table 2. MPE 20 µg/ml has the highest viability cell in all glucose concentration compared to control and other treatments. This data indicated that MPE 20 µg/ml has the highest activity to increase cells proliferation in glucose-induced mesangial cells at 10 days of incubation.

The viability of glucose-induced SV40 MES 13 cells treated with MPE (5, 20 μg/ml) and AM (20, 80 μM) during 15 days of incubation time can be seen in Table 3. In each glucose concentration, the highest cell proliferation was MPE 20 µg/ml in glucose concentration 5 and 25 mM with each value of 113.38±5.10% and 97.85±0.58%. This indicated that MPE 20 µg/ml in 5 and 25 mM glucose concentration is potential to increase cell proliferation in glucose-induced mesangial cells at 15 days of incubation. Based on statistical analysis, MPE has significant difference in elevation of cell proliferation in glucose-induced mesangial cells compared to positive control (*P*<0.05).

***TGF-β1 level***

The reduction of TGF-β1 level in glucose-induced mesangial cells as positive control, glucose-induced mesangial cells treated with MPE (5 and 20 μg/ml) and AM (20 and 80 μM) can be seen in Figure 1. Figure 1 showed the concentration of TGF-β1 by ELISA method after treating glucose-induced SV40 MES 13 cells with MPE and AM. The lowest level of TGF-β1 was obtained in 5 μg/ml of MPE (66.30 pg/ml) at 5 mM glucose-induced, while AM was at 80 μM (65.42 pg/ml) at 5 mM glucose-induced compared to positive control (5 mM glucose-induced) (95.58 pg/ml). Based on statistical analysis, MPE 5 μg/ml has the most significant difference in inhibition of TGF-β1 level of glucose-induced mesangial cells compared to positive control but almost comparable with negative control (*P*<0.05).

***Fibronectin level***

The reduction of fibronectin level in glucose-induced mesangial cells as positive control, glucose-induced mesangial cells treated with MPE (5 and 20 μg/ml) and AM (20 and 80 μM) can be seen in Figure 2. Figure 2 showed the concentration of fibronectin level by ELISA method after treating glucose-induced SV40 MES 13 cells with MPE and AM. The lowest fibronectin level was obtained at 5 μg/ml of MPE (1.11 ng/ml) at 5 mM glucose-induced and 80 μM of AM (2.15 ng/ml) at 5 mM glucose-induced compared to positive controls (5 mM glucose-induced) (7.34 ng/ml). Based on statistical analysis, MPE 5 and 20 μg/ml has the most significant difference compared to positive control but almost comparable compared to negative control (*P*<0.05).

## Discussion

Hyperglycemia is believed to play a pivotal role for the initiation of pathological process. The primary injury is believed to take place in the glomerular tuft and leads to an eventual decline in renal function (28). According to Qian *et al. *(2014), excessive amount of extracelullar glucose leads to glucose uptake in mesangial cells which further leads to an activation of a number of metabolic pathways that results in increased production of reactive oxygen species (ROS) (29) and advanced glycation end products (AGEs). Thus pathways induce ECM production such as fibronectin and critically TGF-β1 synthesis. Yet, overexpression of TGF-β and fibronectin closely linked to glomerulosclerosis (30).

In this study, mesangial cells were cultured in a high concentration of glucose (hyperglicemic) which correlates to diabetic glomerulosclerosis condition. TGF-β and fibronectin was used as a parameter due to their existence in glomerulosclerosis disease. In line with previous study conducted by Nahman *et al. *(1992), there were significant decreasing of cell number after 5, 10 and 15 days incubation in high concentration of glucose (5, 25, and 125 mM). The results suggest that high concentration of glucose may have suppressive effect on mesangial cell proliferation. In addition, the results showed the effect of glucose on cell proliferation is dose dependent (25). The longer the incubation and higher the glucose concentration, the ability of the cells to improve itself is lower. In other study, it also suggested that a high glucose in mesangial cell reduced cell number caused by free radical damage and enhanced ECM (31). Yet, the mechanisms of how glucose inhibit cell proliferation is remain unclear but the production of metabolic waste products unique to a high glucose environment and ROS may contribute to the observed decrease in celullar proliferation (25). 

MPE and AM was used in respect of high level of antioxidants. The pericarps of *G. mangostana *L. is known for its high concentration of xanthones and it has pharmacological effect as antioxidant (19). Under hyperglycaemic conditions, we suggest that antioxidants are able to regenerate a damaged ECM and improve cell growth as a result of oxidative stress through non-enzymatic glycation of proteins (32, 33). Because oxidative stress is associated with glomerulosclerosis and other disease related to a reduced antioxidant defense, therefore, it can be postulated that the antioxidants, which can reduce the oxidative stress and prevent the progression of the disease, may exert a key role to protect mesangial cells in glomerulosclerosis (34, 35). 

Present study shows, that MPE and AM increase cell proliferation and significantly reduced the level of TGF-β and fibronectin in glucose-induced mesangial cells compared to positive control. According to Jha *et al.* (2016) (36), antioxidants are able to convert ROS into nonreactive oxygen molecules which is harmless to cells (36). It also has an effect on retarding glucose absorption through inhibition of carbohydrate-hydrolyzing enzymes such as α-glucosidase and α-amylase (37) and down- regulates the TGF-β expression and fibronectin level (38) by decreasing NADPH oxidase expression. The expression of NADPH oxidase is elevated in diabetic nephropathy and it a source of oxidative stress. The up-regulation of NADPH oxidase subunits p47^phox ^and p22^phox^ plays an important role in ROS production and elevation fibronectin in high glucose condition (39). Antioxidant contained in MPE and AM may also ameliorate the antiproliferative response of mesangial cell to high level of glucose by altering gene transcription factors that act to regulate the cell growth (31).

According to Dennis & Witting (2017), anti-inflammatory agents may potentially reduce ROS via stabilizing endothelium function and NO bioactivity. Thus pathway may improve renal function and decrease tubular damage. Moreover, anti-inflammatory, as well as up-regulating gene responses, linked to antioxidant and cytoprotection (40). Down-regulation of TGF-β1 and fibronectin level indicate an improvement of cell proliferation and metabolism in mesangial cells. This is presumably due to the influence of xanthone and α-mangostin contained in MPE which is able to neutralize free radicals that retarding cell damage (19, 23, 41). 

## Conclusion

Mangosteen peel extract (MPE) (in 5 and 20 μg/ml) increased proliferation of cells in range glucose-induced concentration of 5-25 mM and significantly reduced TGF-β1 and fibronectin levels in glucose-induced mesangial cells in glucose-induced concentration 5-10 mM. In conclusion, MPE products performed glucose induced-mesangial cells as *in vitro* model of diabetic glomerulosclerosis.
